# Une cause rare du choc septique chez le diabétique: la cystite emphysémateuse compliquée d'une rupture vésicale

**DOI:** 10.11604/pamj.2015.20.415.6757

**Published:** 2015-04-27

**Authors:** Abdelkarim Shimi, Abderrahman Boumedian, Nabil Elbakouri, Ali Derkaoui, Mohammed Khatouf

**Affiliations:** 1 Service de Réanimation Polyvalente A1, CHU Hassan II, Faculté de Médecine et de Pharmacie, Université Sidi Mohamed Ben Abdallah, Fez, Maroc

**Keywords:** Diabète, cystite emphysémateuse, tomodensitométrie, choc septique, Diabetes, emphysematous cystitis, TDM, septic shock

## Abstract

La cystite emphysémateuse est une affection rare rencontrée principalement chez les patients diabétiques mal équilibrés. Elle se caractérise par la présence de l'air dans la paroi et/ou la lumière vesicale. La tomodensitométrie abdomino-pelvienne reste l'examen clé pour confirmer le diagnostic. Nous rapportons l'observation d'une patiente diabétique ayant présentée une cystite emphysémateuse favorisée par un diabète déséquilibré et compliquée d'une rupture vésicale intrapéritonéale avec évolution défavorable.

## Introduction

La cystite emphysémateuse (CE) est une affection rare. Elle se caractérise par la présence de gaz dans la lumière et /ou la paroi vésicale. Elle représente une complication d'origine infectieuse secondaire à une pullulation microbienne aéro-anaérobie. Cette affection prédomine chez les femmes diabétiques d’âge moyen, avec parfois un pronostic sévère [1]. Nous décrivons le cas d'une patiente ayant présenté une cystite emphysémateuse dans un contexte de diabète déséquilibré et compliqué d'une rupture vésicale.

## Patient et observation

**Figure 1:**
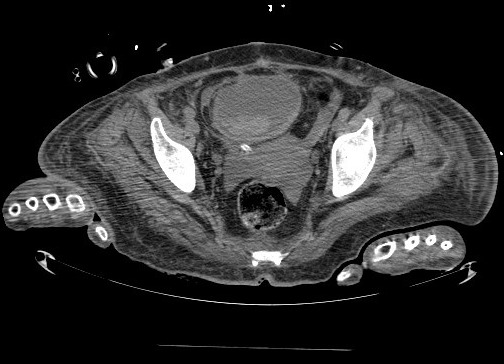
Coupe axiale d'une tomodensitometrie abdomino-pelvienne montrant la présence de l'air au niveau de la paroi vesicale

Patiente âgée de 60 ans; suivie pour diabète de type II depuis 10 ans sous antidiabétique oraux; admise initialement au service d'endocrinologie pour prise en charge d'un diabète déséquilibré sur infection urinaire à E. Coli. 48 heures après; la patiente a présenté des douleurs abdomino-pelviennes; avec vomissements et troubles de conscience dans un cadre de syndrome fébrile d'ou son admission en Réanimation. L'examen clinique a trouvé une patiente confuse; sans déficit sensitivomoteur; fébrile à 39^°^C; la pression artérielle à 130/60 mm Hg; la fréquence cardiaque à 125 battement /mn; notre patiente était polypnéique avec une FR à 22 cycle /minute et la SPO2 à 95% à l'air ambiant. L'examen abdominal a trouvé un abdomen souple avec une légère sensibilité pelvienne. Le sondage urinaire a ramené 350 ML d'urine franchement purulente; l'examen des urines a l'aide de bandelettes a objectivé un taux de sucre à 3 + + + et d'acétone à + +. Le bilan biologique a objectivé une glycémie à 3,8g/l; un taux d'hémoglobine a 11g/dl; des plaquettes à 144 000/mm^3^; hyperleucocytoses à 22 600/mm^3^, le taux de prothrombine à 87%; la protéine C Réactive à 160 mg/ l, avec une insuffisance rénale fonctionnelle (urée à 0,70 g/l et la créatinine à 9mg/ l). Le scanner cérébral était normal, par contre la tomodensitométrie abdomino-pelvienne sans injection (Figure 1) a mis en évidence la présence de l'air dans la paroi de la vessie, avec un épanchement intra péritonéal de faible abondance. Le diagnostic d'une cystite emphysémateuse dans un contexte de diabète déséquilibré a été retenu. La prise en charge a consisté en une réhydratation par le sérum salé isotonique 0.9%; insulinothérapie à la seringue électrique à la dose de 0.1 UI/kg/H et un traitement antibiotique à base de céftriaxone et métronidazole.

**Figure 2:**
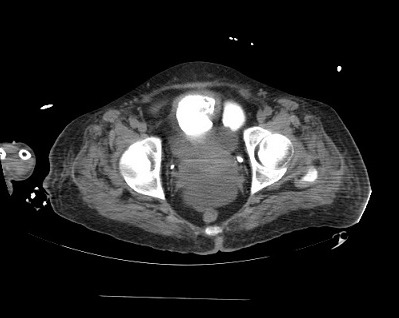
Coupe axiale d'une tomodensitometrie abdomino-pelvienne après opacification vesicale montrant le passage du produit de contrast en intraperitonéal

L’évolution a été marquée par l'altération de l’état clinique de la patiente: persistance de syndrome fébrile avec un taux de globules blancs qui est passé de 22 600/mm^3^ à 31 000/mm^3^; et un taux de CRP qui est passé à 235mg/l; une instabilité hémodynamique nécessitant le recours aux vasopresseurs; apparition d'une distension abdomino-pelvienne d'aggravation progressive. Devant ce tableau clinique une échographie abdomino-pelvienne a été faite et a mis en évidence un épanchement abdomino-pelvien de grande abondance faisant suspecter une perforation vesicale. Une TDM abdomino-pelvienne après opacification vésicale (Figure 2) a montré le passage de produit de contraste en intra péritonéale confirmant le diagnostic de la rupture vésicale. La patient a été admise au bloc opératoire, l'exploration chirurgicale a objectivé un épanchement intra- abdominal de 3 litres d'urines avec nécrose de 3 cm au niveau du dôme vesicale associée à une perforation faisant a peu prêt 1 cm, la patiente a bénéficié d'une nécrosectomie et d'une réparation vésicale en deux plans, associé à un drainage vésical et un lavage péritonéal abondant avec réalisation des prélèvements bactériologiques. Dès la sortie du bloc opératoire, la patiente a installé un choc septique réfractaire avec acidose métabolique sévère (PH à 7,01 HCO3 à10). Malgré une antibiothérapie à large spectre et une réanimation bien conduite; le décès est survenu 24 heures après dans un tableau de dysfonction multiviscérale.

## Discussion

La cystite emphysémateuse (CE) est une infection rare et sévère du bas appareil urinaire. Elle est définie par la présence de l'air au niveau de la paroi et, parfois dans la lumière vésicale. Elle complique les infections urinaires, particulièrement chez les patients diabétiques (60 à 70% des cas) [2]. De plus, la majorité des patients atteints sont de sexe féminin [2, 3], et sont âgés de plus de 60 ans. Les principaux facteurs prédisposant sont un diabète déséquilibré, une stase urinaire vesicale (vessie neurologique, hypertrophie prostatique), une dénutrition, un état d'immunosuppression et une infection urinaire chronique. Le diabète demeure cependant le premier facteur favorisant et reste incriminé dans 60% des cas [4, 5]. La présence de l'air dans les voies urinaire a été décrite pour la première fois en 1671, chez un patient présentant une pneumaturie. Puis en 1961, Bailey a rapporté, sur des autopsies humaines et animales, les premiers cas de cystite emphysémateuse [6]. Sur le plan physiopathologique, la présence de l'air dans la paroi vésicale serait la conséquence d'une production bactérienne de CO_2_. En effet, la formation de gaz résulte de la fermentation du glucose en dioxyde de carbone et hydrogène sous l'action combinée d'un PH acide et d'une glycosurie [3, 7, 8]. Ce processus de fermentation est majoré par la stase urinaire et la déshydratation qui exposent à l'ischémie et à la mauvaise oxygénation tissulaire [5, 8]. L'albumine dans les urines peut également servir de substrat pour les organismes pathogènes [9], enfin l'atteinte vasculaire (sténose des artères rénales et néphro-angiosclérose) altère la défense du tissu hôte contre les organismes pathogènes.

Le diagnostic de la CE est radiologique, la tomodensitométrie est l'examen le plus sensible, et permet d’éliminer une fistule vésico-colique et rechercher une pyélonéphrite associée [10]. La présentation clinique est variée: il existe des douleurs dans 80% des cas et moins souvent, dans 50% des cas des signes vésicaux irritatifs (dysurie, brulures). La CE peut être asymptomatique (7% des cas), ou être révélée par une pneumaturie (7 à 10% des cas) [2]. Les agents bactériens le plus fréquemment retrouvés [3] sont: Escherichia coli (58%); Klepsiella pneumoniae (21%); Enterobacter aerogenes (7%); clostridium perfringens (6%). Le pronostic des CE est le plus souvent favorable, mais peut évoluer vers une pyélonéphrite emphysémateuse avec un risque de choc septique. Il existe également un risque de nécrose de la paroi, pouvant entrainer sa rupture. Dans notre cas, le tableau clinique est celui d'une cystite emphysémateuse chez une patiente qui présente un déséquilibre diabétique sur une infection urinaire encours de traitement. La survenue secondaire de la rupture vésicale et l’évolution défavorable peut être expliquée par un retard diagnostique ou une antibiothérapie inadéquate. La précocité du diagnostic, la qualité et la rapidité du traitement sont les principaux facteurs pronostiques. Tout retard diagnostique et thérapeutique peut conduire à une extension vers les uretères ou le parenchyme rénal. Les complications de la CE sont la cystite nécrosante, la pyélonéphrite emphysémateuse, la septicémie et l'emphysème sous cutané [5, 8, 11]. L’évolution vers la rupture vésicale est exceptionnelle même en l'absence d'un drainage vésicale. De rares cas de rupture vésicale associée à une péritonite ont été décrits [12, 13]. Le traitement est le plus souvent médical. Il consiste en une bi-antibiothérapie à large spectre par voie intraveineuse, associé à un drainage vésical par pose de sonde à demeure. La durée de traitement est mal définie et dépend de la réponse clinique [4, 10]. elle est de 3 à 6semaines. Le traitement chirurgical est parfois nécessaire en cas d’évolution défavorable avec atteinte nécrosante, l'on a alors recours à une cystectomie totale ou partielle.

## Conclusion

Le diagnostic de la cystite emphysémateuse, peut parfois être difficile surtout chez les patients âgés et chez les diabétiques mal équilibrés qui n'expriment pas les symptômes de la douleur. Chez ces patients le recours à l'imagerie est nécessaire, surtout la tomodensitométrie qui reste l'examen clé pour affirmer le diagnostic et évaluer la sévérité des lésions, et rechercher une atteinte rénale associée.
